# Crystal structure of ErmE - 23S rRNA methyltransferase in macrolide resistance

**DOI:** 10.1038/s41598-019-51174-0

**Published:** 2019-10-10

**Authors:** Alena Stsiapanava, Maria Selmer

**Affiliations:** Department of Cell and Molecular Biology, Uppsala University, BMC, Box 596, SE-751 24 Uppsala, Sweden

**Keywords:** Enzymes, Antimicrobial resistance, X-ray crystallography

## Abstract

Pathogens often receive antibiotic resistance genes through horizontal gene transfer from bacteria that produce natural antibiotics. ErmE is a methyltransferase (MTase) from *Saccharopolyspora erythraea* that dimethylates A2058 in 23S rRNA using S-adenosyl methionine (SAM) as methyl donor, protecting the ribosomes from macrolide binding. To gain insights into the mechanism of macrolide resistance, the crystal structure of ErmE was determined to 1.75 Å resolution. ErmE consists of an N-terminal Rossmann-like α/ß catalytic domain and a C-terminal helical domain. Comparison with ErmC’ that despite only 24% sequence identity has the same function, reveals highly similar catalytic domains. Accordingly, superposition with the catalytic domain of ErmC’ in complex with SAM suggests that the cofactor binding site is conserved. The two structures mainly differ in the C-terminal domain, which in ErmE contains a longer loop harboring an additional 3_10_ helix that interacts with the catalytic domain to stabilize the tertiary structure. Notably, ErmE also differs from ErmC’ by having long disordered extensions at its N- and C-termini. A C-terminal disordered region rich in arginine and glycine is also a present in two other MTases, PikR1 and PikR2, which share about 30% sequence identity with ErmE and methylate the same nucleotide in 23S rRNA.

## Introduction

The ribosome is the large macromolecular machine responsible for the sequential template-dependent polymerization of amino acids into a polypeptide chain^1^, and an important drug target for antibiotics^2,3^. Macrolide antibiotics and their third-generation ketolide derivatives are used against a broad range of Gram-positive pathogens. They inhibit translation by binding in the nascent peptide exit tunnel close to the peptidyl transferase center of the large ribosomal subunit^4,5^. One important mechanism of microbial resistance to macrolides is the N6 methylation of 23S rRNA nucleotide A2058 (*Escherichia coli* numbering) in the macrolide binding site by the Erm (erythromycin ribosome methylation) group of MTases^5,6^. This group of enzymes uses SAM to specifically mono- or dimethylate a 50S ribosomal precursor substrate^7,8^, where A2058 is accessible for modification. *Erm* genes were originally identified in microorganisms producing natural macrolides as a mechanism of self-protection against their own antibiotics^9^. ErmE (EC 2.1.1.184) is a dimethyltransferase from the actinomycete *Saccharopolyspora erythraea*, from which the first macrolide antibiotic erythromycin was originally extracted^10,11^. ErmE provides resistance to macrolide, lincosamide, and streptogramin B (MLS) antibiotics to *S. erythraea*^12^. However, in conditions of wide use of antibiotics, horizontal gene transfer has led to propagation of pathogens carrying this and other *erm* genes.

Ketolides were initially developed to overcome macrolide resistance^13^ and present a very promising class of antibiotics^14^. Most members of this class are synthetic and semi-synthetic derivatives of macrolides. However, *Streptomyces venezuelae* strain ATCC 15439 produces the natural ketolide pikromycin^15^. To avoid self-inhibition, this microorganism expresses two MTases PikR1 and PikR2 that mono- and dimethylate A2058^16^, the same nucleotide as ErmE. PikR1 and PikR2 display 39% sequence identity to each other and 35% and 33% sequence identity to ErmE.

In light of the danger of horizontal transfer of macrolide and ketolide resistance genes, there is an urgent need for better understanding of the respective resistance mechanisms, including information on the structural and functional properties of ribosome-modifying enzymes. Here, we present the crystal structure of rRNA MTase ErmE, and analyze the similarities and differences to PikR1, PikR2, ErmC’ and other similar MTases.

## Materials and Methods

### DNA constructs, protein expression and purification

All codon-optimised N-terminally His_8_-tagged constructs were synthesized by GenScript and subcloned into the pET-24a(+) vector (Supplementary Table S1).

Plasmids were transformed into *E. coli* BL21(AI). Cultures were grown at 37 °C in LB media with 0.025 mg/ml kanamycin and 0.1% (w/v) D-glucose until an OD600 of 0.6. Protein expression was induced with 0.1% (w/v) L-arabinose. After overnight cultivation at 18 °C, the cells were collected by centrifugation, resuspended in lysis buffer (50 mM phosphate buffer pH 8, 1 M NaCl and 2 mM ß-mercaptoethanol) supplemented with 10 mM imidazole, 10% (v/v) glycerol, 0.06 mg/ml DNAse and cOmplete protease inhibitor cocktail (Roche, Switzerland), and lysed in a high-pressure homogenizer (Constant System Ltd, UK). The lysate was centrifuged for 1 h at 30,000 g and the supernatant was applied to a gravity-flow column containing Ni-sepharose resin (GE Healthcare, Sweden) equilibrated with lysis buffer and 10% (v/v) glycerol. The column was washed with lysis buffer containing 20 and 30 mM imidazole and protein elution was performed with 500 mM imidazole in 50 mM phosphate buffer pH 8, 0.3 M NaCl and 2 mM ß-mercaptoethanol. Eluted protein was dialysed against 20 mM Tris-SO_4_ pH 8, 0.8 M (NH_4_)_2_SO_4_, 2 mM ß-mercaptoethanol and loaded on a 5 ml HiTrap Phenyl HP column (GE Healthcare, Sweden) equilibrated with dialysis buffer. Elution was done with a linear gradient of (NH_4_)_2_SO_4_ (0.8–0 mM) in 20 mM Tris-SO_4_ pH 8. Size-exclusion chromatography (SEC) was performed using a HiLoad 16/600 Superdex 75 pg column (GE Healthcare, Sweden) equilibrated with running buffer (20 mM Tris-SO_4_ pH 8, 0.3 M (NH_4_)_2_SO_4_ and 2 mM ß-mercaptoethanol). Peak fractions were analysed with SDS-PAGE and concentrated to 10 mg/ml using a 10 kDa cutoff Vivaspin Turbo concentrator (Sartorius, Germany). Purification was performed at 4 °C.

Differential scanning fluorimetry (DSF)^17^ was done using a BioRad CFx connect real time PCR machine.

### Crystallization, data collection and structure determination

All proteins were subjected to sitting drop vapor diffusion crystallization using a mosquito crystallization robot (TTP Labtech, UK). Rhomboid-shaped tetragonal crystals of truncated ErmE grew in 5 d at room temperature in drops of 200 nl in 2% (v/v) tacsimate pH 5.0, 0.1 M sodium citrate tribasic dihydrate pH 5.6 and 16% (w/v) PEG 3350 (PEG/Ion screen, Hampton Research, US). For data collection at beamline ID30A-3 (MASSIF-3)^18^ of the European Synchrotron Radiation Facility (Grenoble, France), the crystal was fished directly from the drop and flash frozen in liquid nitrogen. X-ray experiments were done at 0.9677 Å wavelength at 100 K.

Data was processed using *XDS*^19^. The structure was solved by molecular replacement with *Phaser*^20^, using as search model an ensemble generated from PDB IDs 1QAM^21^, 3FUU^22^, 1YUB^23^, 3FYC^24^ and 1ZQ9 by *CCP4* online pipeline *MrBump*^25^. The structure was traced with *PHENIX AutoBuild*^26^ followed by completion of missing regions in *ARP/wARP*^27^. Manual rebuilding was done in *Coot*^28^ and refinement with *phenix.refine*^29^. Protein geometry was validated in *MolProbity*^30^. All figures representing structures were made using *PyMOL*^31^.

Data collection and refinement statistics are reported in Table 1. A stereo image of a section of the *2mFo-Dfc* map is presented in Supplementary Fig. S1.

**Table 1:** Data collection and refinement statistics. Values in parentheses are for highest-resolution shell.

Data collection	
No. of crystals	1
Space group	P4_3_2_1_2
Cell dimensions	
*a*, *b*, *c*; Å	76.04, 76.04, 104.92
α, β, γ; °	90, 90, 90
Resolution, Å	37.55–1.75 (1.81–1.75)
R_merge_^‡^	0.093 (3.79)
R_pim_^§^	0.025 (0.98)
I/σI^¶^	19.04 (0.77)
Wilson B factor, Å^2^	34.15
Total reflections	474,706 (48,958)
Unique reflections	31,708 (3,116)
Completeness, %	99.91 (99.84)
Redundancy	15.0 (15.7)
CC(1/2)^#^	1 (0.324)
**Refinement**	
Resolution, Å	37.55–1.75
Reflections	31,695 (3,112)
Free reflections	1,829 (180)
R_work_^ll^/R_free_^**^	0.1920/0.2185
Ramachandran plot	
Favored, %	98.76
Allowed, %	1.24
Outliers, %	0.00
No. of atoms	
Protein	2,039
Ligand	5
Water	212
B-factors	
Protein	42.87
Ligand	84.84
Water	42.37
R.m.s deviations	
Bond lengths, Å	0.004
Bond lengths, Å	0.59
PDB ID code	6NVM

^‡^R_merge_, Σ_hkl_Σ_i_|I_i_(hkl) − 〈I(hkl)〉|/Σ_hkl_Σ_i_ Ii(hkl), where I_i_(hkl) is the intensity for an observation of a reflection and 〈I(hkl)〉 is the average intensity of all symmetry-related observations of a reflection.

^§^R_pim_, Σ_hkl_ √(1/n − 1) Σ_i_|I_i_(hkl) − 〈I(hkl)〉|/Σ_hkl_Σ_i_ I_i_(hkl).

^¶^I/σI, signal to noise ratio.

^#^CC(1/2), percentage of correlation between intensities from random half-datasets.

llR_work_, Σ_hkl_||F_obs_| − k|F_calc_||/Σ_hkl_|F_obs_|.

**R_free_, as R_work_, calculated from the free reflections excluded from refinement.

## Results and Discussion

### PikR1, PikR2 and ErmE purification and analyses

After initial purification tests, DSF measurements showed that the thermal stability of PikR1 increased in presence of phosphate and sulphate. For this reason, phosphate buffer was used during lysis and Tris-SO_4_ was used after IMAC, to avoid formation of salt crystals during crystallization.

**Figure 1:**
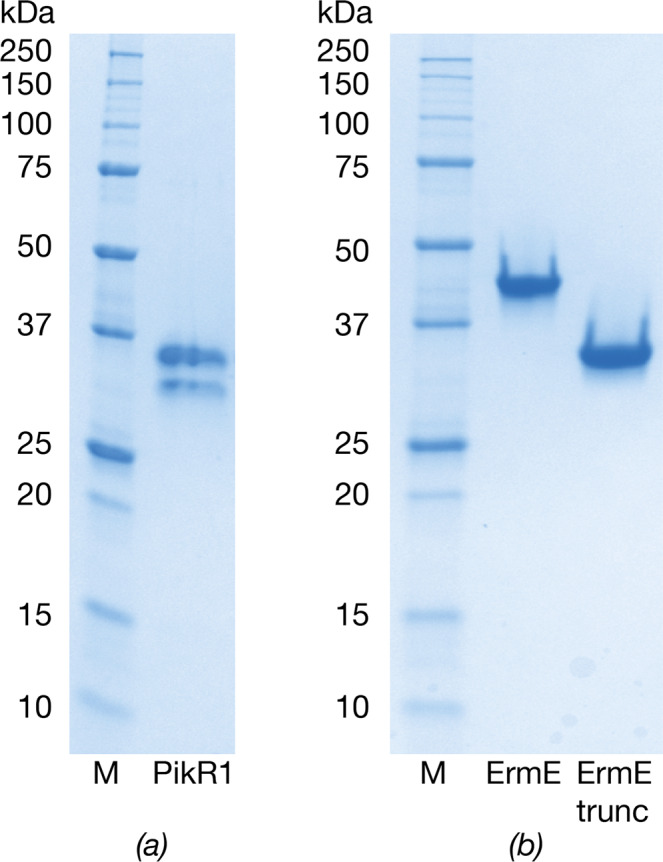
Coomassie-stained SDS-PAGE. (**a**) PikR1 after HIC purification. (**b**) Full-length and truncated ErmE after SEC purification. M: Precision Plus Dual Color Standard (BioRad). Full-length gels are presented in Supplementary Fig. S2.

**Figure 2:**
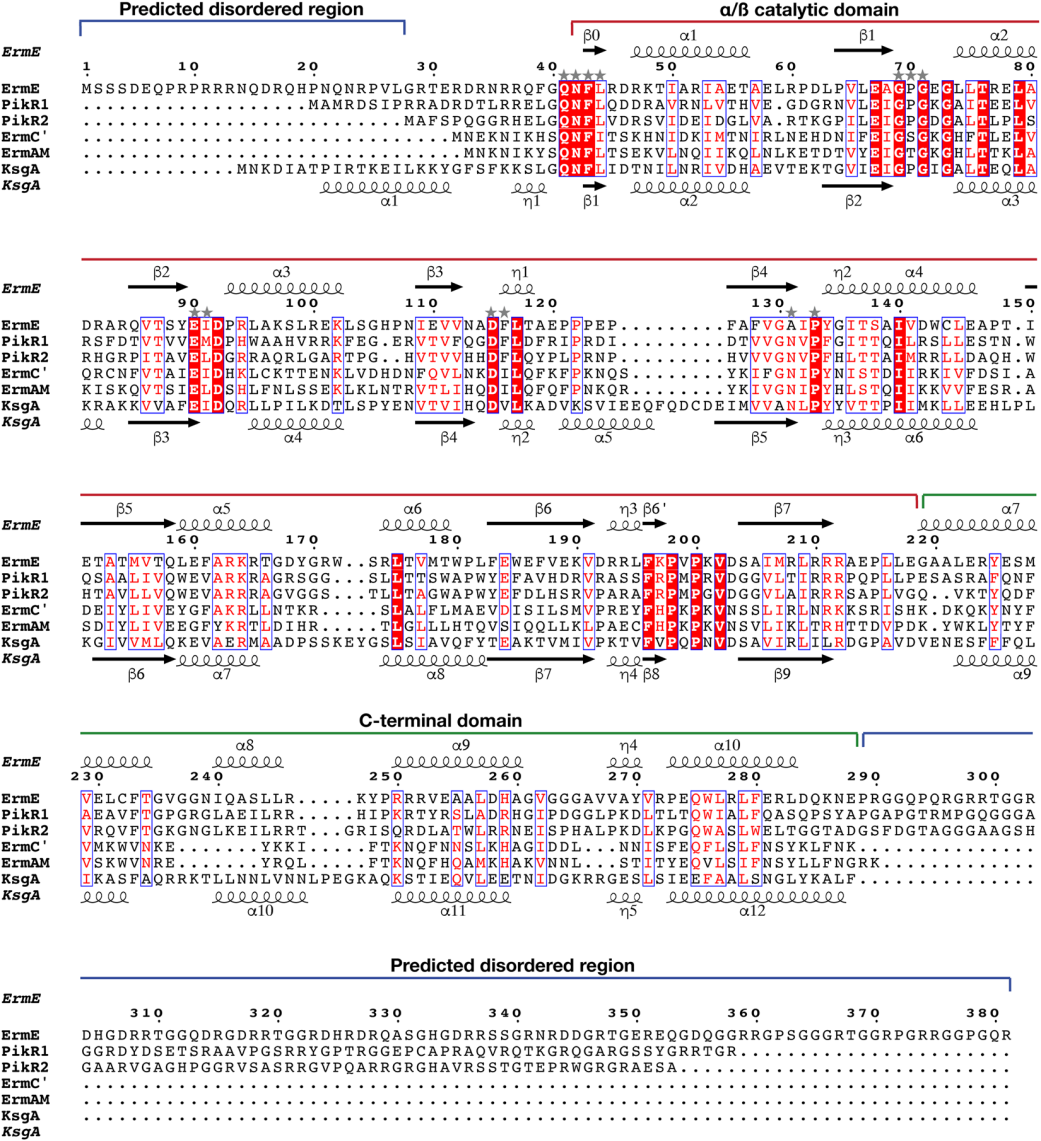
Structure-guided sequence alignment of ErmE with PikR1, PikR2, ErmC’ (PDB 1QAM)^21^, ErmAM (PDB 1YUB)^23^ and KsgA from *B. subtilis* (PDB 6IFT)^52^. Domain organization and secondary structure elements of ErmE are shown above the alignment and secondary structure of KsgA below the alignment. Conserved residues are highlighted with white text on red background and conservative substitutions are presented by red text on white background. Residues predicted to interact with SAM are indicated with stars. The alignment was done with *Expresso*^53^ and visualized with *ESPript 3.0*^54^. Disordered regions of ErmE were predicted with *PrDOS*^32^.

Full-length PikR1 purified using IMAC and HIC was analysed by SDS-PAGE and reproducibly showed two distinct bands (Fig. 1a). To determine the content of the bands and to exclude the presence of another protein, the two bands were subjected to mass-spectrometry analysis at the Proteomics Core Facility at University of Gothenburg (Sweden). The results demonstrated that both bands consisted of PikR1. Since the second, smaller, band was present after IMAC purification of the N-terminally His_8_-tagged PikR1, we hypothesized that it was the result of a C-terminal proteolytic degradation. In support of this, investigation of the PikR1 sequence with the *PrDOS* online server^32^ predicted disorder of a C-terminal region of around 67 aa, which could make the protein susceptible to proteolytic degradation as well as prevent crystallization of the full-length protein. For PikR2 and ErmE, C-terminal regions of 64 and 93 residues were similarly predicted to be disordered (Fig. 2).

Based on this observation, full-length and C-terminally truncated variants of PikR1, PikR2 and ErmE were expressed, purified and subjected to crystallization experiments. Only the truncated version of ErmE (Fig. 1b) produced diffraction-quality crystals.

### Overall structure of ErmE

Crystals of the N-terminally His_8_-tagged construct of C-terminally truncated ErmE (UniProt ID P07287) including residues 1–290 (Supplementary Table S1) diffracted to 1.75 Å and belonged to space group P4_3_2_1_2 with one molecule per asymmetric unit. The structure was solved by molecular replacement using an ensemble of structures with rRNA N6A-methylating activities. The refined structure includes residues 42–285 of ErmE. The absence of ordered density for the N- and C-termini confirms the predicted flexibility of these regions. SEC analysis and examination of the structure in PDBe *PISA*^33^ confirm that ErmE is a monomer.

**Figure 3:**
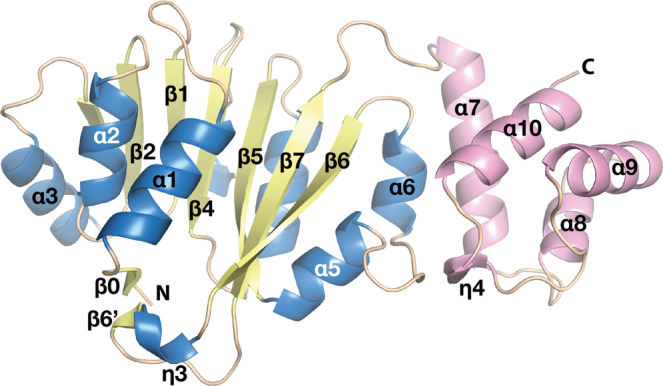
Cartoon representation of overall structure of ErmE. α-helices and ß-strands of the N-terminal α/ß catalytic domain are colored in blue and yellow; α-helices of the C-terminal domain are in pink and loops in wheat.

**Figure 4:**
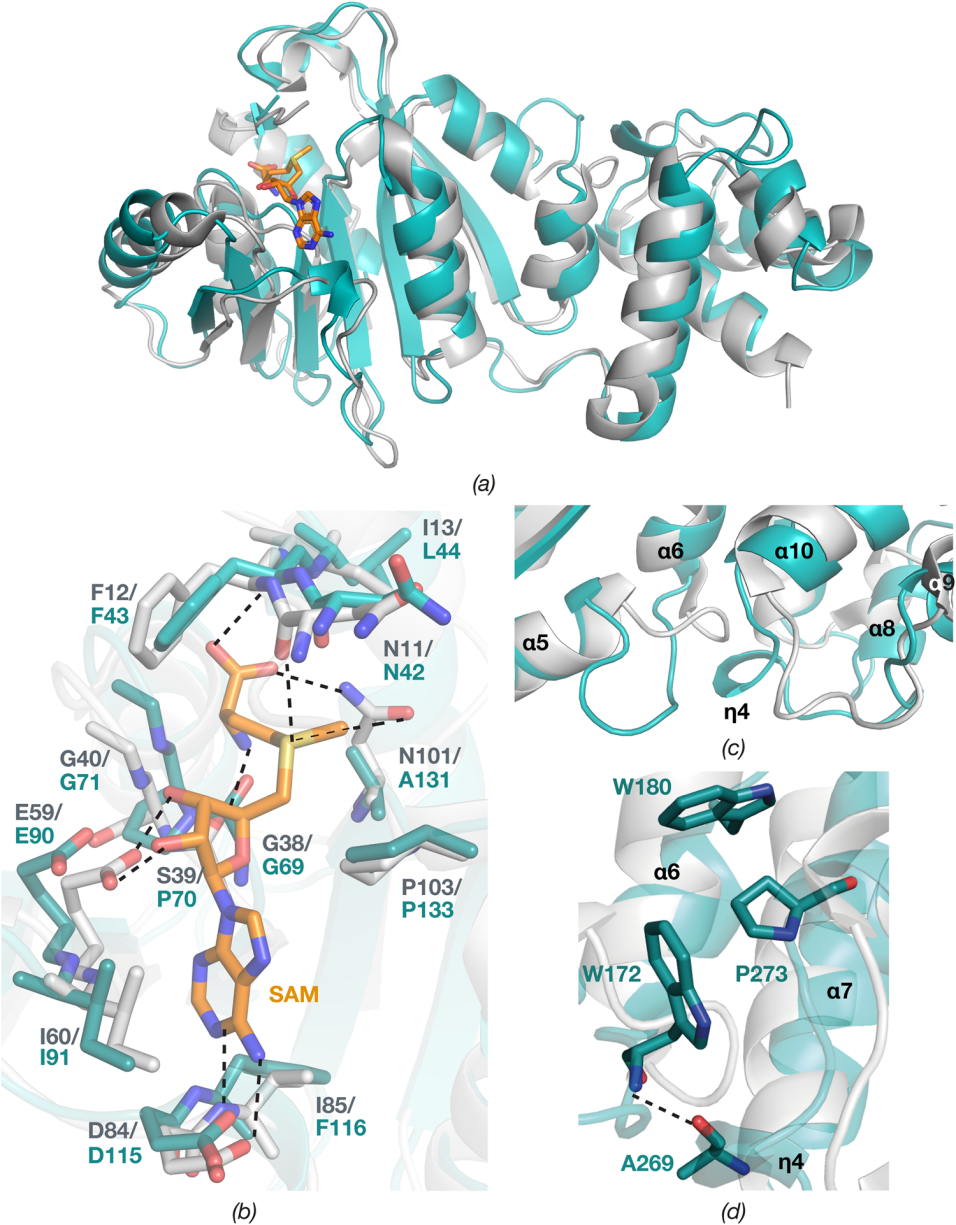
Superposition of ErmC’ (grey) in complex with SAM (orange) (PDB ID 1QAO)^21^ onto ErmE (teal). (**a**) Overall structures. (**b**) Residues interacting with SAM at the SAM binding site. Main chain carbonyl oxygens are only shown if involved in SAM binding. Hydrogen bonds are shown by dashed lines. (**c**) Comparison of interdomain region including the α5-α6 and α9-α10 loops and the additional η4 in ErmE. (**d**) Residues involved in interdomain interactions in ErmE.

The bilobed structure of ErmE consists of an N-terminal Rossmann-like α/ß catalytic domain (residues 42–211) and C-terminal helical domain (residues 219–285), which are connected by a loop (Fig. 3). *DALI*^34^ identified dimethyltransferase ErmC’ (EC 2.1.48) from *Bacillus subtilis* (PDB ID 1QAM)^21^ as the structure most similar to ErmE, with root mean square deviation (rmsd) of 2.75 Å over 230 C^α^ atoms of the superposed structures (Fig. 4a). Interestingly, ErmE and ErmC’ share only 24% sequence identity despite having the same function and modifying the same site in 23S RNA. Other similar structures identified by *DALI* are 16S rRNA A1518 and A1519 MTase KsgA (PDB ID 3FUV, rmsd 2.42 Å over 216 C^α^ atoms)^22^ and its archaeal homologue Dim1 (PDB ID 3FYC, rmsd 2.70 Å over 219 C^α^ atoms)^24^. Since these enzymes modify a different RNA substrate, we decided to mainly compare the ErmE structure to the structure of ErmC’.

The Rossmann-like fold is common for nucleotide-binding proteins in general^35^, and the most common fold of the catalytic domain of SAM-dependent MTases^36^. The C-terminal domain was in ErmC’ proposed as an RNA-binding domain^37^ based on its large positively charged surface. However, it was later shown by mutagenesis that the key residues for specific RNA binding are located in the catalytic domain, facing the cleft between the domains. Accordingly, the C-terminal domain was suggested to mainly function in structural stabilization of the catalytic domain^38^.

### N-terminal catalytic domain

The catalytic domain consists of seven parallel (ß1-ß6 and ß6’) and two antiparallel (ß0 and ß7) ß-strands that are surrounded by three α-helices (α1-α3) and one 3_10_ helix (η3) on one side, and three α-helices (α4-α6) and two 3_10_ helices (η1-η2) on the opposite side (Figs 2 and 3).

ErmE and ErmC’ have the same topology of their catalytic domains (Fig. 4a) that share 26% sequence identity. The domains superpose with rmsd of 1.65 Å over 155 C^α^ atoms, the main difference being a longer loop between helices α5 and α6 in ErmE.

**Figure 5:**
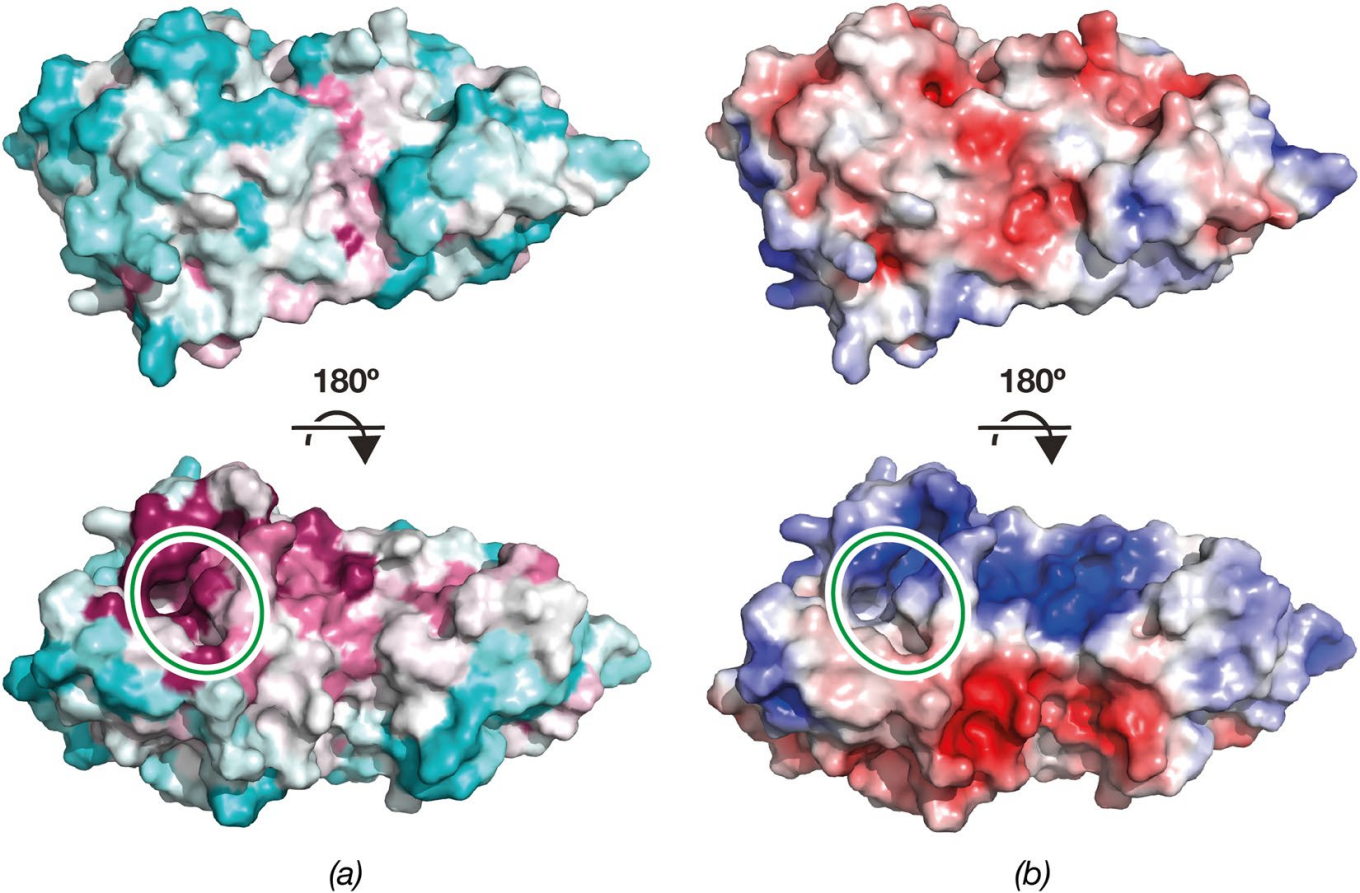
Surface representation of ErmE. (**a**) ErmE surface colored according to sequence conservation calculated by *ConSurf*^55^. The color spectrum ranges from magenta (highest conservation) to cyan (lowest conservation). (**b**) ErmE surface colored by electrostatic potential. The color spectrum ranges from deep red (−5 kT) to deep blue (+5 kT). The SAM binding site is indicated by a green ellipse.

The N-terminal domain shows an L-shaped pocket rich in conserved residues (Figs 4a and 5). Conserved areas containing positively charged residues are also found above and to the right of the pocket (Fig. 5a,b), suggesting that these regions are involved in binding of the rRNA substrate.

### SAM binding site

Superposition of the catalytic domains of ErmE and ErmC’ in complex with SAM suggests that SAM will bind similarly in the lower part of the pocket, with the methyl group directed towards the upper part of the pocket (Fig. 4a,b), where the substrate adenine will likely bind. Analysis of the ErmE structure with the *3DLigandSite* server^39^ predicts the same binding site for SAM. Most SAM-binding residues are conserved from ErmC’, with minor differences presented by Ile13/Leu44 and Ser39/Pro70, involved in main-chain interactions with SAM, and Ile85/Phe116, where the side chain makes a hydrophobic interaction with the adenine group of SAM (Fig. 4b). Interestingly, the substitution of Asn101 in ErmC’ with Ala131 in ErmE suggests that the carboxyl group of SAM will only be coordinated through a hydrogen bond to the main chain N of Leu44, while in ErmC’ the same carboxyl in addition hydrogen bond to Asn101 N^δ2^.

These residues are part of the sequence motif IV^40^_131_AIPY_134_ in ErmE and _101_NIPY_104_ in ErmC’ (Fig. 2), that is observed for example in dimethylating RNA or DNA N6-MTases (consensus sequence (A/S/N)(L/I/V)P(Y/F)^41^). Intriguingly, PikR1 that was reported to be a monomethylating MTase^16^, instead of the (N/D)PP(Y/F/W) motif associated with monomethylating N6A-MTases, contains the same NVPF motif as the dimethyltransferase PikR2 (Fig. 2) and both proteins are assigned to the same Pfam^42^ family of RNA dimethylases (PF00398).

### C-terminal domain

In ErmE, the C-terminal domain is built from four α-helices (α7-α10) and one 3_10_ helix (η4) (Figs 2 and 3). The C-terminal domains of ErmE and ErmC’ have similar topology, and despite only 18% sequence identity superpose with a rmsd of 1.78 Å over 53 C^α^ atoms. Thus, the longer α8 in ErmE corresponds to the short η4 in ErmC’. Interestingly, ErmC’ has a deletion at the position of the FTG tripeptide in α7 of ErmE that is conserved in both PikR MTases (Fig. 2). Another feature of ErmE is a longer loop with an inserted η4 helix between α9 and α10 (Fig. 3), where ErmC’ only contains a shorter loop (Fig. 4c).

Together with the C-terminal loop, η4 participates in interactions with the loop between α5 and α6 of the catalytic domain, contributing to stabilising the structure of ErmE (Fig. 3). A hydrogen bond is formed between main chain atoms of Ala269 and Trp172 and a hydrophobic interaction between Pro273, Trp172 and Trp180 (Fig. 4d). In ErmC’, the corresponding interaction is different due to the absence of η4 (Fig. 4c), and the involved residues are not conserved (Fig. 2).

In addition, the difference in interactions between N- and C-terminal domains in ErmE and ErmC’ leads to the slightly different orientation of these domains relative to each other, which results in a higher rmsd value for the superposition of the whole MTase structures as compared to when the individual domains are superposed.

### Recognition of substrate RNA

In addition to ErmE, ErmC’ and ErmAM that provide antibiotic resistance, structures are available of two bacterial rRNA N6A-MTases involved in ribosome biogenesis, KsgA^43^ and RlmJ^44^ and catalytic domains of the human mRNA N6A-MTases METTL3-METTL14^45^ and METTL16^46–48^. These enzymes all display similar structures of their catalytic domains but make use of a variety of loops, tails or extra domains for specific recognition of the sequence and structure of their respective RNA substrates. There are few structures of N6A MTases in complex with RNA; KsgA^49,50^ and METTL16^47^. KsgA methylates a close to mature 30S subunit and similarly to the Erm family of MTases contains a C-terminal helical domain. For ErmE, it has been shown that although the natural substrate is a precursor of the 50S ribosomal subunit, the enzyme can specifically methylate a 27-nucleotide stem loop RNA substrate mimicking the local environment of A2058^51^. Thus, some essential recognition elements in the RNA are located in close proximity to the adenosine that is methylated.

On the protein side, mutational studies on ErmC’ showed that a single arginine in equivalent position to Lys164 in α5 of ErmE is essential for erythromycin resistance^38^. In the ErmE structure, a sulfate ion is bound between Lys164 and Arg174, possibly mimicking a substrate phosphate. The positively charged surface of the C-terminal domain is also likely to contribute to substrate binding.

The N-terminal disordered region of ErmE is rich in arginine, while the C-terminal disordered region is dominated by glycine and arginine. Predicted disordered low-complexity regions with similar characteristics are also present in PikR1 and PikR2. These regions may contribute to binding of the 50S ribosome assembly intermediate where A2058 is accessible for modification. Similarly to the positively charged tails of ribosomal proteins, they may order upon interaction with the negatively charged RNA backbone. However, ErmC’ does not contain the corresponding long tails but can still recognize and modify the same substrate (Fig. 2).

Recently, KsgA was engineered to alter its substrate specificity and allow activity on the Erm substrate^52^. The strategy was based on exchanging the C-terminal domain, the N-terminal tail including α1 and η1 and the loop between α7 and α8 in KsgA to the corresponding sequences from ErmC’. The structure-guided sequence alignment of ErmE with ErmC’, ErmAM and KsgA (Fig. 2) shows that the Erm family enzymes, despite methylating the same substrate, display large variation in sequence and length in the N-terminus and the α7- α8 loop (KsgA numbering). This suggests that, out of the exchanged regions, the C-terminal domain is the more characteristic sequence element for the MTases that display specificity for each RNA substrate.

## Conclusions

Here, we present the first crystal structure of rRNA methyltransferase ErmE, determined at 1.75-Å resolution. The structure of the enzyme could be potentially used for structure-based drug design with the aim to prevent macrolide antibiotic resistance in pathogens. Considering its higher than 30% sequence identity to PikR1 and PikR2, the structure of ErmE is also expected to be useful as a molecular replacement search model for further studies of PikR MTases.

## Supplementary information

Supplemental material

## Data Availability

Atomic coordinates of ErmE have been deposited in the Protein Data Bank with accession code 6NVM.
