# Supplementary material for: Crystal structure of ErmE - 23S rRNA methyltransferase in macrolide resistance
Source: Sci Rep. 2019 Oct 10;9:14607. doi: 10.1038/s41598-019-51174-0 (PMC6787224; doi:10.1038/s41598-019-51174-0)
Supplement: Supplementary file 1 — Supplemental material [file 41598_2019_51174_MOESM1_ESM.pdf]

Supplemental material for

Crystal structure of ErmE - 23S rRNA methyltransferase in macrolide resistance

Alena Stsiapanava* and Maria Selmer*

Department of Cell and Molecular Biology, Uppsala University, BMC, Box 596, SE-751 24 Uppsala, Sweden

* to whom correspondence should be addressed: alena.stsiapanava@gmail.com or maria.selmer@icm.uu.se

Content: Supplementary Table S1
Supplementary Figure S1-S2

Supplementary Table S1

Details of protein expression constructs

	PikR1 (UniProt ID: Q9ZGI6)	PikR1 truncated
Source organism	*S. venezuelae* strain ATCC 15439	
Expression vector	pET-24a(+)	
Expression host	*E. coli* BL21(AI)	
Complete amino-acid sequence	MHHHHHHHHMAMRDSIPRRAD RDTLRRELGQNFLQDDRAVRNL VTHVEGDGRNVLEIGPGKGAITE ELVRSFDTVTVVEMDPHWAAHV RRKFEGERVTVFQGDFLDFRIPR DIDTVVGNVPFGITTQILRSLLES TNWQSAALIVQWEVARKRAGRS GGSLLTTSWAPWYEFAVHDRVR ASSFRPMPRVDGGVLTIRRRPQP LLPESASRAFQNFAEAVFTGPGR GLAEILRRHIPKRTYRSLADRHGI PDGGLPKDLTLTQWIALFQASQP SYAPGAPGTRMPGQGGGAGGRD YDSETSRAAVPGSRRYGPTRGGE PCAPRAQVRQTKGRQGARGSSY GRRTGR	MHHHHHHHHMAMRDSIPRRAD RDTLRRELGQNFLQDDRAVRNL VTHVEGDGRNVLEIGPGKGAITE ELVRSFDTVTVVEMDPHWAAHV RRKFEGERVTVFQGDFLDFRIPR DIDTVVGNVPFGITTQILRSLLES TNWQSAALIVQWEVARKRAGRS GGSLLTTSWAPWYEFAVHDRVR ASSFRPMPRVDGGVLTIRRRPQP LLPESASRAFQNFAEAVFTGPGR GLAEILRRHIPKRTYRSLADRHGI PDGGLPKDLTLTQWIALFQASQP SYALE
	**PikR2 (UniProt ID: Q9ZGI7)**	**PikR2 truncated**
Source organism	*S. venezuelae* strain ATCC 15439	
Expression vector	pET-24a(+)	
Expression host	*E. coli* BL21(AI)	
Complete amino-acid sequence	MHHHHHHHHMAFSPQGGRHEL GQNFLVDRSVIDEIDGLVARTKG PILEIGPGDGALTLPLSRHGRPITA VELDGRRAQRLGARTPGHVTVV HHDFLQYPLPRNPHVVVGNVPF HLTTAIMRRLLDAQHWHTAVLL VQWEVARRRAGVGGSTLLTAG WAPWYEFDLHSRVPARAFRPMP GVDGGVLAIRRRSAPLVGQVKT	MHHHHHHHHMAFSPQGGRHEL GQNFLVDRSVIDEIDGLVARTKG PILEIGPGDGALTLPLSRHGRPITA VELDGRRAQRLGARTPGHVTVV HHDFLQYPLPRNPHVVVGNVPF HLTTAIMRRLLDAQHWHTAVLL VQWEVARRRAGVGGSTLLTAG WAPWYEFDLHSRVPARAFRPMP GVDGGVLAIRRRSAPLVGQVKT

	YQDFVRQVFTGKGNGLKEILRRT<br>GRISQRDLATWLRRNEISPHALP<br>KDLKPGQWASLWELTGGTADGS<br>FDGTAGGGAAGSHGAARVGAG<br>HPGGRVSASRRGVPQARRGRGH<br>AVRSSTGTEPRWGRGRAESA	YQDFVRQVFTGKGNGLKEILRRT<br>GRISQRDLATWLRRNEISPHALP<br>KDLKPGQWASLWELTGGTADGS<br>LE

	**ErmE (UniProt ID: P07287)**	**ErmE truncated**
Source organism	*S. erythraea*	
Expression vector	pET-24a(+)	
Expression host	*E. coli* BL21(AI)	
Complete amino-acid sequence	MHHHHHHHHMSSSDEQPRPRRR<br>NQDRQHPNQNRPVLGRTERDRN<br>RRQFGQNFLRDRKTIARIAETAE<br>LRPDLPVLEAGPGEGLLTRELAD<br>RARQVTSYEIDPRLAKSLREKLS<br>GHPNIEVVNADFLTAEPPPEPFAF<br>VGAIPYGITSAIVDWCLEAPTIET<br>ATMVTQLEFARKRTGDYGRWSR<br>LTVMTWPLFEWEFVEKVDRRLF<br>KPVPKVDSAIMRLRRRAEPLLEG<br>AALERYESMVELCFTGVGGNIQ<br>ASLLRKYPRRRVEAALDHAGVG<br>GGAVVAYVRPEQWLRLFERLDQ<br>KNEPRGGQPQRGRRTGGRDHGD<br>RRTGGQDRGDRRTGGRDHRDRQ<br>ASGHGDRRSSGRNRDDGRTGER<br>EQGDQGGRRGPSGGGRTGGRPG<br>RRGGPGQR	MHHHHHHHHMSSSDEQPRPRRR<br>NQDRQHPNQNRPVLGRTERDRN<br>RRQFGQNFLRDRKTIARIAETAE<br>LRPDLPVLEAGPGEGLLTRELAD<br>RARQVTSYEIDPRLAKSLREKLS<br>GHPNIEVVNADFLTAEPPPEPFAF<br>VGAIPYGITSAIVDWCLEAPTIET<br>ATMVTQLEFARKRTGDYGRWSR<br>LTVMTWPLFEWEFVEKVDRRLF<br>KPVPKVDSAIMRLRRRAEPLLEG<br>AALERYESMVELCFTGVGGNIQ<br>ASLLRKYPRRRVEAALDHAGVG<br>GGAVVAYVRPEQWLRLFERLDQ<br>KNEPRLE

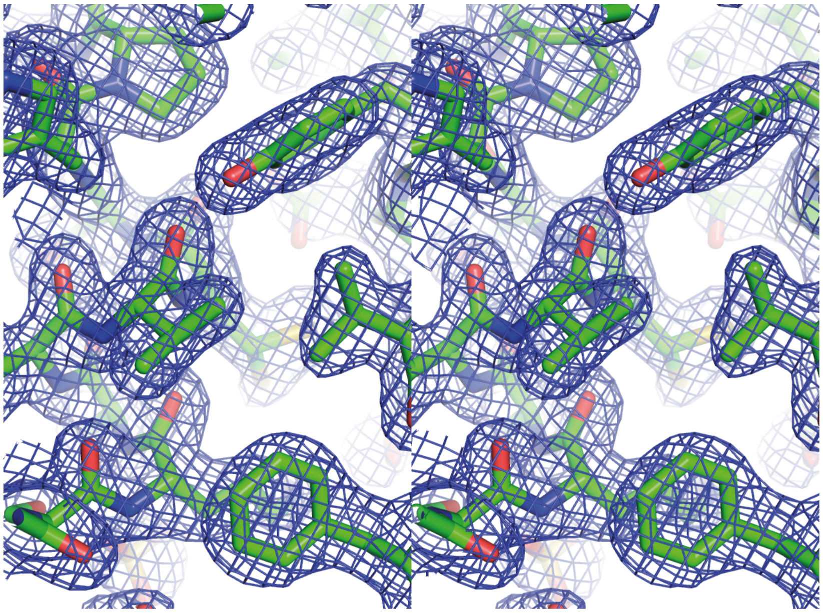

Supplementary Figure S1

Walleye stereo map of ErmE (PDB ID 6NVM) is superimposed onto the corresponding atomic model, shown in stick representation. *2mFo-Dfc* map is contoured at 1.0 σ and centered around V177.

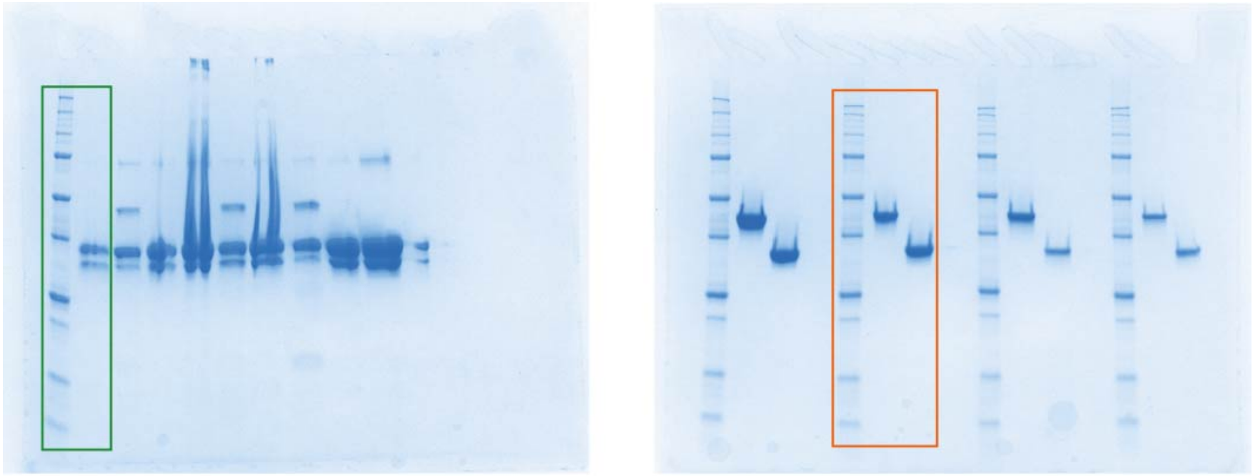

(a) (b)

Supplementary Figure S2

Coomassie-stained SDS-PAGE. *(a)* PikR1 after HIC purification. (*b*) Full-length and truncated ErmE after SEC purification.
